# Takotsubo cardiomyopathy in a patient with ileus: a case report

**DOI:** 10.1186/s12872-017-0700-5

**Published:** 2017-10-17

**Authors:** Chen-Yu C. Guo, Nan-Sung Chou

**Affiliations:** 1 Lewis Katz School of Medicine, Temple University, 3500 N. Broad Street, Philadelphia, PA 19140 USA; 2 Department of Surgery, Madou Sin-Lau Hospital, 20 Lingzilin, Tainan, 72152 Taiwan

**Keywords:** Takotsubo cardiomyopathy, Ileus, Stress, Ventricular dilatation, Ventriculography, Case report

## Abstract

**Background:**

Takotsubo cardiomyopathy (TCM) is a form of stress-induced cardiomyopathy featured by the dilatation of the apex of the left ventricle during systole. Whereas the pathogenesis of this disorder is not well understood, it usually occurs after an emotional or physical stress such as acute asthma, surgery, chemotherapy, and stroke. However, its occurrence in ileus patients is rarely reported. We hereby report probably the first case of TCM after ileus in the literature and discuss its implications.

**Case presentation:**

An 85-year-old man was brought to the Emergency Department due to vomiting, abdominal pain, and no stool passages for 2 days. His abdomen was markedly distended, and ileus pattern was observed in the plain film of abdomen. Electrocardiogram showed right axis deviation, poor R-wave progression, and diffuse ST-segment elevation in the anterior leads, and cardiomegaly was observed by roentgenogram. A ventriculography showed an ejection fraction of 33% and confirmed the apical dilation consistent with TCM. He was treated with medication and discharged without remarkable adverse events. A follow-up transthoracic echocardiogram 4 months later showed normalization of his left ventricular systolic functions.

**Conclusion:**

The precise mechanisms of the development of TCM are still unknown, but it is widely believed that it is triggered by the catecholamine surge produced in response to stress. This case demonstrated that such a stress can be of various forms, including ileus and other conditions that may lead to severe abdominal pain, and highlight the importance of awareness in diagnosing this rare but potentially lethal condition.

## Background

Takotsubo cardiomyopathy (TCM) is a form of stress-induced cardiomyopathy, featured by the dilatation of the apex of the left ventricle during systole. It was first described in the English language in 1991 [1] and was given the name because of the dilatation of the left ventricular apex that leads to the appearance of a Japanese octopus trap (takotsubo) [2]. The pathogenesis of this disorder is not well understood, but a preceding emotional or physical stress is a unique feature, with approximately two-thirds of cases having associated identifiable acute stressors [3–5]. Such physical stressors include acute asthma, surgery, chemotherapy, and stroke [6]. However, to our knowledge, its occurrence in ileus patients has not been reported. We hereby report a case of TCM in a patient with paralytic ileus and discuss its implications.

## Case presentation

**Fig. 1:**
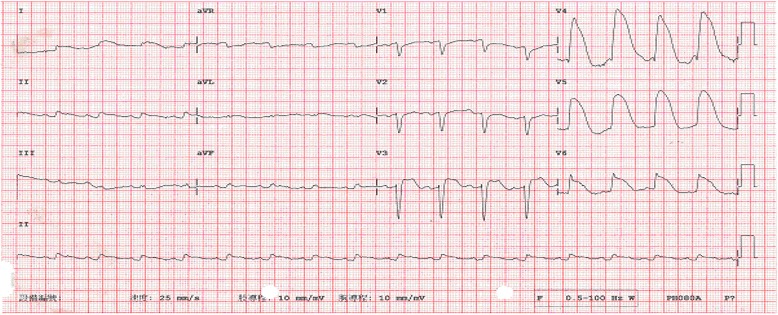
The 12-lead electrocardiogram shows ST-segment elevation in the anterior leads, in association with prolonged QT intervals

**Fig. 2:**
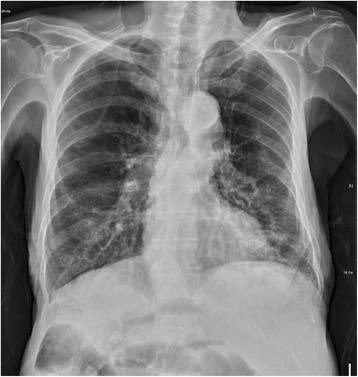
Prominent left heart border without marked cardiomegaly

**Fig. 3:**
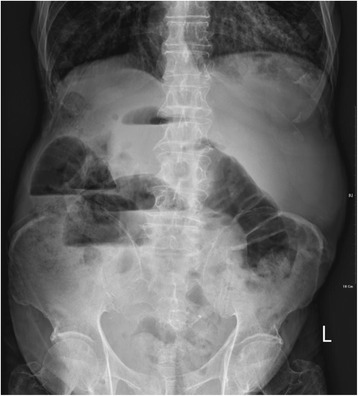
Multiple air-fluid levels suggesting bowel obstruction

An 85-year-old man with diabetes, hypertension, and chronic obstructive pulmonary disease was brought by ambulance to the Emergency Department with the chief complaints of vomiting, abdominal pain, and no stool passages for 2 days. Physical examination revealed labored breathing. Patient also demonstrated peritoneal signs, including muscle guarding and rebounding tenderness. Bowel sounds were absent, but cardiac sounds were normal without rub, murmur, or gallop. Upper and lower extremity pulses were intact and symmetric. His initial electrocardiogram (ECG) showed normal sinus rhythm with right axis deviation, poor R-wave progression, and diffuse ST-segment elevation in the anterior leads (Fig. 1). A roentgenogram demonstrated marked cardiomegaly (Fig. 2). Laboratory data revealed a hemoglobin level of 11.7 g/dL, a hematocrit level of 34.5%, a potassium level of 4.2 mEq/L, a creatine kinase level of 342 U/L, a creatine kinase MB fraction of 23.7 ng/mL, a relative index of 13.2%, and a troponin I level of 8.10 ng/mL. Results of the initial arterial blood gas analysis with the use of 10 L nonrebreathing mask were as follows: pH, 7.30; carbon dioxide tension, 45 mmHg; oxygen tension, 75 mmHg; base excess, −4; and oxygen saturation, 90%. On presentation, the patient’s vital signs and cardiopulmonary examination were normal. His abdomen was markedly distended, and ileus pattern was illustrated in the plain film of abdomen (Fig. 3).

**Fig. 4:**
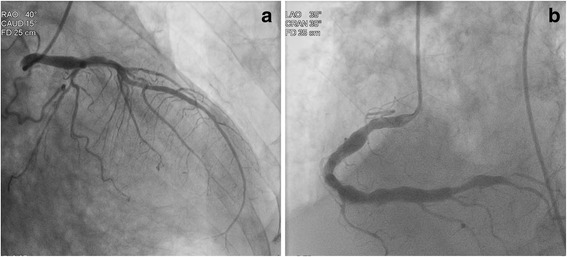
Coronary angiography of the patient showing no significant obstructions: (**a**) left coronary angiography and (**b**) right coronary angiography

**Fig. 5:**
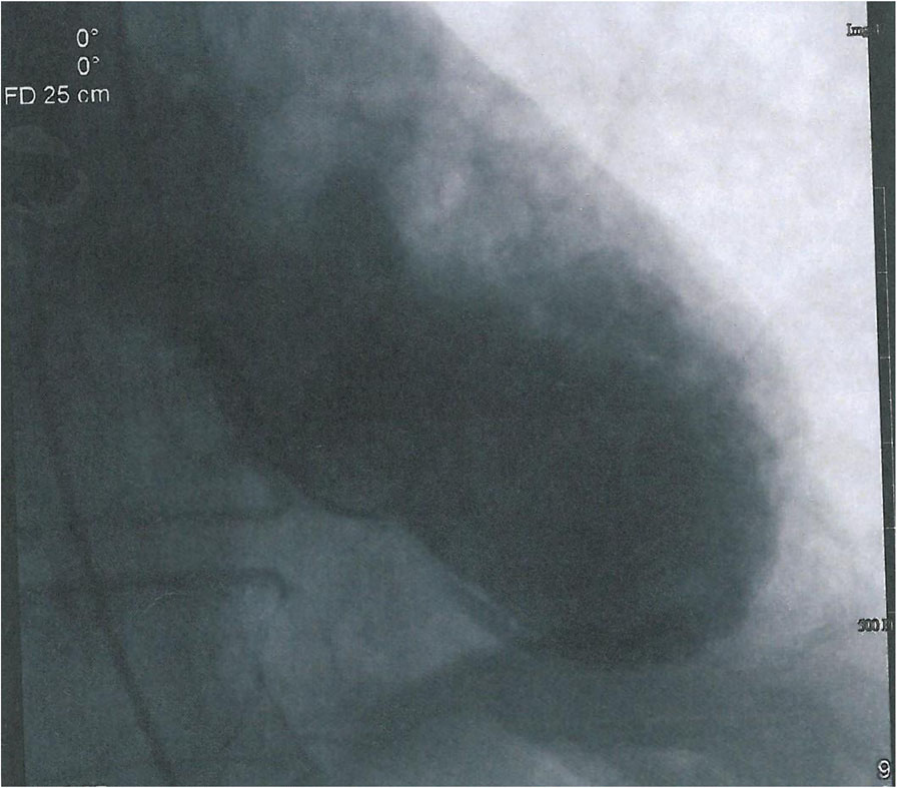
Left heart catheterization showing persistent left ventricle apical hypokinesis with systolic ballooning

As laparotomy was suggested by a surgeon who was consulted in the intensive care unit, the patient received pre-anesthesia assessment. On the basis of the recommendation from a cardiologist who performed the assessment, the patient underwent left heart catheterization on the second day of hospitalization, which failed to show a significant obstruction in any coronary distribution (Fig. 4). Ventriculography estimated an ejection fraction of 33% and confirmed apical dilation consistent with TCM (Fig. 5). The patient was medically managed and discharged without adverse events. A follow-up transthoracic echocardiogram 4 months later showed normalization of his left ventricular systolic function.

The CARE guidelines were followed in this report.

## Discussion and conclusions

Over 90% of TCM cases are observed in postmenopausal women [7], and clinical manifestations of TCM may range from asymptomatic ECG abnormalities and nonspecific systemic symptoms, to heart failure, cardiogenic shock, and sudden death. We report an uncommon case of a male patient with TCM accompanied by paralytic ileus. The diagnosis of TCM in this patient was confirmed by the elevated troponin I level (8.10 ng/mL), elevated ST-segment with prolonged QT interval, and unique cardiac imaging that showed left ventricular ballooning. Although the precise mechanisms behind TCM are unclear, it is generally believed that this disorder is triggered by the catecholamine surge produced during the body’s sympathetic response to stress. This is evidenced by the fact that a preceding emotional or physical stress is a unique feature of TCM, with approximately two-thirds of cases having associated, identifiable acute stressors [3–6]. Elevated catecholamine levels and reversible left ventricle ballooning have also been observed in a rat model of immobilization-induced stress [8], and cases of pheochromocytoma with the presentation of TCM confirmed through ventriculography or echocardiography have been reported [7, 9, 10]. Patients with TCM were found to have a higher prevalence of neurologic and psychiatric disorders [11], and psychopathological traits such as neuroticism, depression, and anxiety may persist even after recovery [12].

The physical stressors that can induce TCM include acute asthma attack, surgery, chemotherapy, stroke, car accident, suicide attempt, etc. [6, 7, 10, 13]. Various gastrointestinal symptoms such as acute cholecystitis, vomiting, and diarrhea are considered possible triggers for TCM [7, 14]. In fact, a review of 3719 patients in Japan found that 57 (1.5%) of them had acute gastrointestinal diseases [14]. However, paralytic or any kind of ileus is not specifically listed as one of them [7]. Furthermore, using “Takotsubo cardiomyopathy” combining “ileus,” “intestine,” “colon,” or “gastrointestinal” as key words to search the literature indexed in PubMed, we did not find any previous reports on cases associated with ileus, while a single report specifically on a diarrhea-related case was found [15]. Because the ileus was resolved without any surgical interventions, many acute disorders and conditions that might act as a stressor triggering TCM in this patient can be generally ruled out. As the patient’s history did not reveal any other remarkable triggers for TCM, we believe that the ileus caused by stool impaction, and the abdominal pain occurred subsequently, acting as a stressor for developing TCM. It is possible that acute reduction of cardiac output due to foregoing TCM deteriorates the bowel movement, which in turn leads to ileus. However, the initial presentation of this patient was ileus, and the typical symptoms of TMC such as chest discomfort were developed after admission to the hospital. In addition, he did not have history of obvious bowel dysfunction before this episode. Therefore, we believe ileus was more likely to be the cause instead of the consequence of TCM in this case.

Given the nonspecific symptoms and signs, a high clinical index of suspicion is essential for prompt diagnosis of TCM, a rare but potentially fatal disorder. One of the indices of suspicion is either emotional or physical stimulus that is stressful for the patient. This case suggests that ileus, and probably severe abdominal pain caused by other etiologies, can be added to the list of stress triggers that can cause TCM and alarm clinicians.

## Abbreviations

EKG: Electrocardiogram
TCM: Takotsubo cardiomyopathy
